# Socio-cultural context as a protective factor: regional disparities in adolescent mental health in Mexico during the COVID-19 pandemic

**DOI:** 10.3389/frcha.2026.1783057

**Published:** 2026-06-22

**Authors:** Rodrigo Ramirez-Rodriguez, Rafael Fernández-Demeneghi, Yuliana Yessy Gomez-Rutti, Sandra Cristina Pillon, Ángel Alberto Puig-Lagunes

**Affiliations:** 1 Instituto Politécnico Nacional, Ciudad de México, Mexico; 2 Instituto de Investigaciones en Comportamiento Alimentario y Nutrición, Universidad de Guadalajara, Ciudad Guzmán, Mexico; 3 Universidad Privada del Norte, Lima, Peru; 4 Escuela de Enfermería de Ribeirão Preto de la Universidad de São Paulo, Ribeirão Preto, Brazil; 5 Facultad de Medicina, Universidad Veracruzana, Minatitlán, Mexico

**Keywords:** drugs, high school students, mental health, psychopathology, resilience

## Abstract

**Background:**

Globally, approximately one in seven adolescents experiences a mental disorder, which can negatively impact their quality of life. In Mexico, evidence of regional differences in adolescent mental health is limited. This study examined regional disparities in the prevalence and severity of mental health symptoms, substance use, and resilience among adolescents in southern Veracruz during the COVID-19 pandemic. Additionally, we identified psychosocial profiles and examined the protective role of resilience against psychopathology.

**Methods:**

A cross-sectional comparative study was conducted among adolescents from four regions of southern Veracruz: semi-urban, agro-commercial, industrial urban, and semi-rural. Participants completed online, self-administered questionnaires assessing psychoactive substance use, symptoms of stress, anxiety, and depression, and resilience. Descriptive analyses, binomial logistic and linear regression models, and k-means clustering were used to examine regional differences and the association between resilience and mental health outcomes. Analyses were performed in RStudio (macOS).

**Results:**

A total of 2,194 adolescents (mean age: 16.6 ± 1.1 years) were included. Overall, 28.3% reported alcohol consumption and 8.5% tobacco use. Anxiety was most prevalent (38.5%), followed by depression (33.7%) and stress (23.9%). Only 21.2% demonstrated high resilience. Significant regional differences were observed. Region 4 adolescents (semi-rural) showed fewer moderate-to-extremely severe cases and higher resilience (*p* < 0.001). Adolescents in Regions 1 and 3 were more likely to experience depression (OR = 1.04) and had fewer protective factors, whereas greater social support and structure were associated with Region 4 (OR = 0.69 and 0.92). Higher resilience was associated with lower depression (R² = 0.098) and anxiety (R² = 0.060). Two profiles were identified: Resilient (56.5%) and Vulnerable (43.5%), with Region 4 overrepresented in the Resilient Profile (66.4%) and Region 3 in the Vulnerable Profile (53.2%).

**Conclusions:**

Adolescents from Region 4 exhibited more favorable emotional profiles, characterized by higher resilience and lower symptom severity. The identification of psychosocial profiles and the protective role of resilience highlight the influence of regional context on adolescent mental health, thereby supporting the development of context-specific interventions.

## Introduction

Adolescence is a critical developmental window marked by profound neurobiological maturation, including structural and functional changes in the reward and stress-response systems of the brain. During this period, the emotional regulation system remains underdeveloped, heightening vulnerability to psychopathology and risk-taking behaviors such as substance use (1). These hormonal, physical, and psychological changes can interfere with optimal development and negatively impact quality of life (2). This inherent vulnerability was severely tested during the COVID-19 pandemic, which acted as a profound chronosystem-level shock.

Prolonged school closures and the rapid transition to remote education caused by the pandemic in Mexico have significantly disrupted the daily routines, academic engagement, and social interactions of adolescents (3, 4). These measures, implemented as part of national mitigation strategies, have been associated with increased psychological distress, limited access to peer support, and greater exposure to adverse home environments, particularly in socioeconomically vulnerable regions (5).

Social distancing measures, disruption of daily routines, and school closures exacerbated psychological symptoms, significantly increasing the mental health burden on adolescents (6, 7). Furthermore, these social restrictions limited peer interactions and access to substances, altering habitual consumption patterns during this period (8, 9).

Globally, approximately one in seven individuals aged 10–19 years experiences a mental disorder, with depression, anxiety, and substance use disorders being the leading causes of illness and disability (10–12). The literature has widely demonstrated that protective factors, both individual and social, play a key role in mitigating emotional distress during adolescence. Resilience, defined as the capacity to adapt to adversity, has been associated with improved emotional regulation and reduced susceptibility to depression and anxiety (13, 14). Similarly, family and social support networks provide emotional security and resources that help adolescents cope with stressors and environmental instability (15). Conversely, adolescent substance use has been consistently associated with emotional dysregulation and mental health issues (16, 17). Therefore, it is important to examine these factors together because they are modifiable psychosocial mechanisms that can mitigate the impact of adverse contextual conditions on adolescents' mental health.

However, while these proximal protective mechanisms are widely recognized, adolescent mental health is also deeply embedded in broader ecological disparities. Economic and social factors, such as family problems, reduced access to essential development resources, a greater frequency of adverse events, reduced perceived social support, and an increased presence of physical and parental health issues, strongly influence mental health trajectories (18–21). Adolescents grappling with mental health challenges often encounter social exclusion, stigmatization, risky behaviors, and poor physical health, which can significantly impact their educational outcomes and family dynamics [(11), Cortés-Flores et al., 2020]. However, the interaction between specific macro-contextual variables, such as regional urbanization, economic structure, and cultural heritage, and these established individual factors in either buffering or exacerbating emotional distress remains poorly understood.

Previous studies have shown that the differences in mental health between urban and rural adolescents are shaped by distinct environmental and structural conditions. Urban environments are consistently linked to an increased risk of mental health issues due to factors like social inequality, population density, and constant exposure to psychosocial stressors (22). In contrast, rural environments may offer protective elements, such as lower environmental stress and greater perceived well-being. However, they are also characterized by limited access to mental health services and support systems (23). Taken together, these findings support the expectation of regional disparities in adolescent mental health. These disparities are not due to one context being universally more vulnerable than the other, but rather due to the distinct patterns of risk and protection that emerge across urban and rural settings.

The southeastern region of Veracruz, Mexico offers a unique geographical and sociocultural landscape for examining these dynamics. This area is characterized by stark regional disparities, juxtaposing highly urbanized, industrial petrochemical hubs with semi-rural, socioeconomically marginalized municipalities that maintain strong Indigenous cultural roots. Despite the critical need for context-specific data, empirical evidence from this region remains limited. However, regional studies have reported that over 40% of adolescents exhibit symptoms of anxiety, depression, and alcohol use (24–29).

To bridge this gap, this study examines regional disparities in mental health symptoms, substance use, and resilience among adolescents across four structurally distinct regions of southeastern Veracruz during the COVID-19 pandemic. Incorporating geographic and sociocultural context, the study aims to describe and compare patterns of vulnerability and protective factors across regions. This approach provides an empirical basis for identifying context-sensitive factors that could inform the development of targeted, community-specific intervention and prevention strategies.

## Methods

A cross-sectional study was conducted on adolescents enrolled in nine public high schools in southern Veracruz, Mexico. This was an analytical and observational study. The participating schools were approached through the regional coordinator of the Colegio de Bachilleres del Estado de Veracruz (COBAEV), who oversees the nine campuses in the southern region included in this study. This centralized structure ensured coordinated institutional authorization and standardized communication with the principals. Participation was voluntary and subject to formal approval by school authorities, as well as coordination of access procedures with the parents or legal guardians. Schools were included based on institutional authorization and logistical feasibility.

The nine participating schools were distributed across four predefined regions: Region 1 included three schools: Cosoleacaque, Chinameca, and Jaltipan. Region 2 included three schools: Acayucan, Sayula de Aleman, and Juan Rodriguez Clara. Region 3 included one school: Minatitlan. Region 4 included two schools: Soteapan and Zaragoza.

Given the well-documented influence of social context on adolescent mental health and substance use, the study population was *a priori* stratified into four regions using objective sociodemographic indicators derived from the 2020 Population and Housing Census conducted by the Instituto Nacional de Estadística y Geografía (30). Regional classification considered geographic proximity, population size, economic structure, level of urbanization, household income, marginalization index, access to health and educational services, and cultural composition of the region. This stratification aimed to enhance internal validity by increasing within-group homogeneity and reducing potential contextual confounding in regional comparisons of anxiety, depression, resilience, and the use of psychoactive substances.

Region 1: Cosoleacaque, Chinameca, and Jaltipán, which are semi-urban areas. The populations of these municipalities were approximately 130,903 in Cosoleacaque, 22,638 in Chinameca, and 38,669 in Jaltipán, reflecting an intermediate population density between urban and rural areas. These municipalities have diverse economic activities, including agriculture, local commerce, and services, influenced by nearby industrial development. Culturally, social life in this region reflects both agricultural traditions and increasing integration with industrial workforces and urban lifestyles due to its proximity to petrochemical and manufacturing hubs.
Region 2: Acayucan, Sayula de Alemán, and Juan Rodríguez Clara are intermediate urban agro-commercial areas. Acayucan has approximately 80,815 inhabitants, Sayula de Alemán has approximately 32,400 inhabitants, and Juan Rodríguez Clara has approximately 38,367 inhabitants. These municipalities share an economy driven mainly by agriculture, livestock, and regional commerce, with a substantial portion of the working population engaged in the primary and tertiary sectors characteristic of intermediate urban centers.
Region 3: Minatitlán is an urban industrial municipality with a significant petrochemical industry and more formal commerce. The municipal seat had approximately 101,336 inhabitants in 2020 and is the core of the Minatitlán metropolitan area, which collectively accounts for over 350,000 inhabitants across several municipalities in Veracruz. Economic activity in this region is driven by the secondary (industrial) and tertiary (services/trade) sectors, with more structured, formal employment than in other regions.
Region 4: Soteapan and Zaragoza (two campues) are semi-rural municipalities with an indigenous presence and a certain degree of marginalization. Soteapan, with approximately 28,104 inhabitants, and Zaragoza, with a smaller population, both have communities where languages such as Popoluca and Náhuatl are spoken alongside Spanish, indicating significant cultural and linguistic diversity. These areas are characterized by lower levels of industrial integration and a higher reliance on subsistence agriculture, with notable rural settlement patterns.

This contextual classification enabled the examination of outcomes within relatively homogeneous socio-structural environments while maintaining meaningful variability between regions. This strengthened the interpretability of the regional differences.

## Participants

We conducted a non-probability sample of approximately 4,380 students from designated secondary schools. All students enrolled in grades 9 through 11 during the February–June 2021 academic semester who were between the ages of 14 and 18 were invited to participate on a voluntary basis.

The inclusion criteria were enrollment in the specified academic period; provision of informed consent for participants aged 18 years or older, or assent for minors; parental or guardian provision of informed consent for participants under 18 years of age; and completion of the questionnaires in full. Students expressed their willingness to participate by selecting the informed consent or assent option at the beginning of the Google Forms questionnaire and answering all items. For participants under 18 years of age, written informed consent was obtained in advance from their parents or legal guardians. Thus, participation required both parental consent and student assent. Students who did not participate were excluded, even if parental consent had been obtained.

Students with a previous psychiatric or psychological diagnosis made by a specialist, as well as those currently receiving treatment, were excluded to minimize possible bias related to ongoing clinical care.

Of the 2,359 adolescents invited to participate, 167 declined, resulting in a final sample size of 2,194 participants. The response rate was 53.85%. Although official data on enrollment in all upper secondary schools by municipality are not available, public education directories indicate that municipalities such as Cosoleacaque, Acayucan, and Juan Rodríguez Clara together have approximately 20 upper secondary schools, while Minatitlán has between 25 and 30. Smaller municipalities, such as Soteapan, Zaragoza, and Chinameca, tend to have one or two upper-secondary schools each.

Therefore, in the regional context, the inclusion of these nine upper secondary schools was designed to capture educational environments representative of the semi-urban, urban, and semirural areas of southern Veracruz.

## Instruments

### Socioeconomic data

The survey included questions about sociodemographic data, such as age, sex, semester, exercise habits, hobbies, the tutor's educational level, and work history outside of the student's home.

### Mexican Resilience Scale (RESI-M)

The RESI-M is a 43-item Likert-type instrument (1 = totally disagree to 4 = totally agree) structured into five dimensions: Strength and Self-Confidence, Social Competence, Family Support, Social Support, and Structure.

Strength and Self-Confidence refer to clarity of goals, perseverance, and confidence when facing challenges (e.g., “I am able to overcome difficult situations”). Social Competence reflects ease in interpersonal relationships (e.g., “I find it easy to establish good relationships with others”). Family Support assesses perceived emotional and practical backing from family members (e.g., “My family supports me when I face problems”). Social Support captures supportive bonds within broader social networks, such as friends or teachers (e.g., “I can rely on my friends when I need help”). Finally, Structure refers to organization and goal-oriented planning (e.g., “I organize my activities to achieve my goals”).

RESI-M scores range from 43 to 172 points. Higher scores indicate greater resilience. The following are the cut-off points: Below 50% is low, 51%–75% is medium, and above 76% is high. This criterion was applied at both the general and dimension-specific levels. The scale has a total consistency of 0.93 according to Cronbach's *α*, which explains 43.6% of the variance. It has been used in an adolescent population (31, 32). Reliability analyses in the present study further confirmed the robustness of the following subscales: strength and self-confidence (Cronbach's *α* = 0.95, McDonald's *ω* = 0.96), social competence (*α* = 0.92, *ω* = 0.92), family support (*α* = 0.93, *ω* = 0.93), social support (*α* = 0.93, *ω* = 0.93), and structure (*α* = 0.88, *ω* = 0.88). These results underscore the measure's reliability in our sample.

### The Alcohol, Smoking and Substance Involvement Screening Test (ASSIST)

The second version of ASSIST, developed by the World Health Organization in 2002, aims to identify licit and illicit substance use, assess associated risks, and determine suitable interventions for individuals. The questionnaire consists of seven questions about each substance, asking about past-life consumption, desire to consume, and resulting problems *(e.g., “In your life, which of the following substances have you ever used?” or “During the past three months, how often have you had a strong desire or urge to use…?”)*. For the purposes of this study, we only considered lifetime consumption. This reliable and valid instrument can be used for research, diagnosis, and intervention with the adolescent population (33, 34).

### The Depression, Anxiety and Stress Scale-21 (DASS-21)

This is a set of three self-report scales designed to measure depression, anxiety, and stress. Each subscale contains seven items that are scored from 0 to 3, and interpreted separately *(e.g., “I felt that I had nothing to look forward to” for depression, “I felt I was close to panic” for anxiety, and “I found it difficult to relax” for stress)*. Scores were calculated by summing the items of each subscale. In line with common practice in DASS-21 studies, raw scores were used without being multiplied by 2. The severities were categorized as follows: normal (0–3), mild (4–5), moderate (6–7), severe (8–9), and extremely severe (≥ 10) (35, 36). This instrument has been psychometrically validated in the Mexican population, demonstrating high reliability (Mislevy and Bock's *α* = 1.00; McDonald's *Ω* = 0.95) (35). In the present study, reliability analyses confirmed the robustness of the subscales (stress: Cronbach's *α* = 0.88, McDonald's *ω* = 0.89; depression: *α* = 0.90, *ω* = 0.903; anxiety: *α* = 0.88, *ω* = 0.88).

### Procedure

The principals of the selected schools were asked to help distribute the questionnaires and encourage parents and students to voluntarily complete them. Students who agreed to participate signed a consent form and completed the questionnaires via Google Forms. As mentioned previously, students under the age of 18 were required to obtain parental consent and their own agreement. Groups were established by region for the purposes of data analysis, taking into account geographic proximity, usage patterns, and socioeconomic development.

### Statistical analysis

Descriptive statistics included frequencies and percentages for categorical variables and means with standard deviations for continuous variables.

Regional differences in substance use and mental health. Predictor selection across regions was performed using binomial logistic regression with automated model selection via the glmulti algorithm. Region 4 (semi-rural) was selected as reference level. Associations were reported as odds ratios (ORs) and 95% confidence intervals.

The Relationship Between Resilience and Psychopathology Simple linear regressions were computed to examine the association between resilience and mental health outcomes. Total resilience scores (RESI-M) were used as the predictor, and depression and anxiety scores (DASS-21) were used as separate outcomes. Regional variables were not included as moderators in these models because the primary objective was to assess the overall association between resilience and mental health outcomes. Regional differences were examined through complementary analyses.

Cluster analysis and validation. To complement variable-centered analyses and identify naturally occurring psychosocial profiles, a k-means cluster analysis was conducted. This analysis included depression and anxiety scores, the five dimensions of the RESI-M (social support, family support, structure, confidence, and social competence), the total resilience score, and lifetime alcohol use. All variables were standardized (z-scores) prior to clustering to ensure equal weighting. The optimal number of clusters was determined using three methods: the elbow method, average silhouette width, and the NbClust procedure, which evaluates 30 indices simultaneously. The final clustering solution was obtained using k-means with 50 random starts and a maximum of 200 iterations. Once the clusters were identified, the participants were assigned to their respective cluster. Descriptive statistics (means and standard deviations) were calculated for all clustering variables per cluster. Independent-samples t-tests (with Welch's correction for unequal variances) were used to compare continuous variables between clusters. Effect sizes were calculated using Cohen's d with a pooled standard deviation and 95% confidence intervals. To examine whether cluster membership varied by geographic region, a chi-square test of independence was performed, and the effect size was reported as Cramer's V.

All statistical tests were two-tailed, with significance defined as *p* < 0.05. Analyses were conducted using RStudio (macOS).

### Ethical considerations

The project was evaluated and approved by the Bioethics Committee for Research at the Faculty of Medicine of the University of Veracruz, Minatitlan Campus (FOLIO: F-001-CI-2022). Informed consent was obtained from participants aged 18 years or older, while assent was obtained from participants under 18 years of age, along with parental or guardian informed consent, in accordance with the Declaration of Helsinki and the General Health Law of Mexico (37). Additionally, data confidentiality was guaranteed in accordance with the Federal Law on the Protection of Personal Data Held by Private Parties, approved by the Chamber of Deputies of Mexico in 2011.

## Results

### Sociodemographic information

The sample included 2,194 students, 58.9% of whom were female and 41.1% male. The mean age of the students was 16.6 ± 1.1 years old. In terms of regional distribution, 37.9% of the adolescents belonged to Region 1 (semi-urban), 21.5% to Region 2 (agro-commercial), 20% to Region 3 (industrial urban), and 26.2% to Region 4 (semi-rural). Most students (39.7%) were in their sixth semester and engaged in sports activities (62.1%). Most students also had hobbies (82.8%), came from nuclear families (63.8%), and had tutors with secondary-level education or lower qualifications (52.9%). Conversely, 13.9% of the adolescents reported working in addition to studying (Table 1).

**Table 1:** Sociodemographic and academic characteristics of participants by region.

Variable	Category	Region 1 (*n* = 833)	Region 2 (*n* = 473)	Region 3 (*n* = 263)	Region 4 (*n* = 575)	Total (*n* = 2,194)	Cramer's V
Sex	Female	548 (62.1)	265 (56.0)	166 (63.1)	314 (54.6)	1,293 (58.9)	0.07*
	Male	335 (37.9)	208 (44.0)	97 (36.9)	261 (45.4)	901 (41.1)	
Semester	2nd	286 (32.4)	129 (27.3)	101 (38.4)	187 (32.5)	703 (32.0)	0.13*
	4th	171 (19.4)	164 (34.7)	86 (32.7)	198 (34.4)	619 (28.2)	
	6th	426 (48.2)	180 (38.1)	76 (28.9)	190 (33.0)	872 (39.7)	
Age (years)	14–15	169 (19.1)	74 (15.7)	58 (22.1)	100 (17.5)	401 (18.3)	0.10*
	16–17	532 (60.2)	291 (61.8)	178 (67.7)	383 (66.8)	1,384 (63.1)	
	≥ 18	182 (20.6)	106 (22.5)	27 (10.3)	90 (15.7)	405 (18.5)	
Occupation	Study only	795 (90.0)	369 (78.0)	250 (95.1)	475 (82.6)	1,889 (86.1)	0.16*
	Study and work	88 (10.0)	104 (22.0)	13 (4.9)	100 (17.4)	305 (13.9)	
Hobby	No	149 (16.9)	95 (20.1)	32 (12.2)	101 (17.6)	377 (17.2)	0.05
	Yes	734 (83.1)	378 (79.9)	231 (87.8)	474 (82.4)	1,817 (82.8)	
Sports practice	No	349 (39.5)	163 (34.5)	117 (44.5)	203 (35.3)	832 (37.9)	0.06
	Yes	534 (60.5)	310 (65.5)	146 (55.5)	372 (64.7)	1,362 (62.1)	
Tutor's education level	Secondary or lower education	419 (47.5)	274 (57.9)	75 (28.5)	393 (68.3)	1,161 (52.9)	0.19*
	High school	292 (33.1)	129 (27.3)	88 (33.5)	116 (20.2)	625 (28.4)	
	University/postgraduate	172 (19.5)	70 (14.8)	100 (38.0)	66 (11.5)	408 (18.5)	

Data are presented as *n* (%). Differences across regions were assessed using the chi-square test. Effect size is reported as Cramer's V. *p* < 0.05. Semi-urban (Region 1), agro-commercial (Region 2), industrial urban (Region 3), and semi-rural (Region 4).

* Significant difference across regions determined by chi-square test (*p* < 0.05).

### Substance use by region

In general, alcohol was the most commonly consumed psychoactive substance (28.3%), followed by tobacco (8.5%). Marijuana (4.2%), inhalants (1.7%), and cocaine (1.4%) were consumed to a lesser extent than alcohol and tobacco. Compared to other regions, adolescents from Region 3 had a higher lifetime alcohol consumption. No differences in the consumption of other substances were observed between the regions (Table 2).

**Table 2:** Distribution of lifetime psychoactive substance use among different regions.

Drug	Category	Region 1 (*n* = 833)	Region 2 (*n* = 473)	Region 3 (*n* = 263)	Region 4 (*n* = 575)	Total (*n* = 2,194)	Crameŕs V
Alcohol	No	609 (69)	329 (69.6)	171 (65)	465 (80.9)	1,574 (71.7)	0.12*
	Yes	274 (31)	144 (30.4)	92 (35)	110 (19.1)	620 (28.3)	
Tobacco	No	801 (90.7)	432 (91.3)	238 (90.5)	536 (93.2)	2,007 (91.4)	0.03
	Yes	82 (9.3)	41 (8.7)	25 (9.5)	39 (6.8)	187 (8.5)	
Marijuana	No	850 (96.3)	445 (94.1)	250 (95.1)	557 (96.9)	2,102 (95.8)	0.05
	Yes	33 (3.7)	28 (5.9)	13 (4.9)	18 (3.1)	92 (4.1)	
Cocaine	No	870 (98.5)	464 (98.1)	262 (99.6)	568 (98.8)	2,164 (98.6)	0.03
	Yes	13 (1.5)	9 (1.9)	1 (0.4)	7 (1.2)	30 (1.3)	
Inhalants	No	866 (98.1)	465 (98.3)	258 (98.1)	568 (98.8)	2,157 (98.3)	0.02
	Yes	17 (1.9)	8 (1.7)	5 (1.9)	7 (1.2)	37 (1.6)	

Data are presented as *n* (%). Differences across regions were assessed using the chi-square test. Effect size is reported as Cramer's V. *p* < 0.05.

* Significant difference across regions determined by chi-square test (*p* < 0.05).

### Psychopathology and resilience between regions

Conversely, anxiety symptoms were the most prevalent psychopathology across all regions, affecting 38.5% of adolescents in all regions. Among this group, 19.1% experienced extremely severe symptoms. Depressive symptoms were observed in 33.7% of the students. Regarding stress, 23.9% of the adolescents exhibited symptoms. Regarding resilience, 30% of the participants exhibited low levels, 48.8% exhibited medium levels, and 21.2% exhibited high levels. Additionally, elevated levels of family and social support were found in 41.2% and 40.6% of the adolescents, respectively. This was followed by elevated levels of strength and self-confidence (35.5%), structure (22.5%), and social competence (19.6%) (Supplementary Table 1).

### Association between mental health indicators and regional context

The results of the predictor model comparing each region against the reference category (Region 4, semi-rural) are presented in Table 3.

**Table 3:** Binomial logistic regression comparing regions (reference = region 4).

Region comparison	Predictor	OR (95% CI)	*p*-value
Region 1 vs. 4	Depression	**1.04** **(1.02–1.06)**	< 0.001
	Resilience	1.01 (0.99–1.03)	0.27
	Social competence	**1.29** (**1.03–1.60)**	0.02
	Social support	**0.79** (**0.65–0.97)**	0.03
	Family support	**1.26** (**1.01–1.58)**	0.04
	Structure	**0.92** (**0.87–0.97)**	< 0.001
Region 2 vs. 4	Depression	1.00 (0.98–1.03)	0.81
	Resilience	0.99 (0.97–1.01)	0.37
	Social competence	1.25 (0.97–1.61)	0.08
	Social support	0.82 (0.65–1.04)	0.1
	Family support	1.25 (0.96–1.62)	0.09
	Structure	1.00 (0.94–1.07)	0.91
Region 3 vs. 4	Depression	**1.06** (**1.03–1.09)**	< 0.001
	Resilience	**1.03** (**1.00–1.05)**	0.02
	Social competence	**1.56** (**1.15–2.12)**	< 0.001
	Social support	**0.63** (**0.47–0.84)**	< 0.001
	Family support	**1.68** (**1.22–2.30)**	< 0.001
	Structure	**0.87** (**0.80–0.94)**	< 0.001

Odds ratios (ORs) were obtained from binomial logistic regression models. Region 4 was used as the reference category. Statistically significant associations (*p* < 0.05) are shown in bold.

The data concerning substance use were excluded from the binomial regression analysis because they did not emerge as relevant predictors during the variable reduction process.

### Region 1 (semi-urban) vs. Region 4

Adolescents in Region 1 had higher depression scores than those in Region 4 (OR = 1.04, *p* < 0.001). A one-unit increase in social competence was associated with a 29% increase in the odds of belonging to Region 1 (OR = 1.29, *p* = 0.02), and a one-unit increase in family support with a 26% increase in odds (OR = 1.26, *p* = 0.04). In contrast, higher social support was associated with a 21% decrease in odds (OR = 0.79, *p* = 0.03), and each additional unit of structure with an 8% reduction in odds (OR = 0.92, *p* < 0.001). Resilience showed no significant association.

### Region 2 (agro-commercial) vs. Region 4

All odds ratios were close to 1 and non-significant, indicating that depression, resilience, social competence, social support, family support, and structure were not associated with differences in the odds of belonging to Region 2 relative to Region 4.

### Region 3 (industrial urban) vs. Region 4

Each one-unit increase in depression was associated with a 6% increase in the odds of belonging to Region 3 (OR = 1.06, *p* < 0.001); resilience with a 3% increase (OR = 1.03, *p* = 0.02); social competence with a 56% increase (OR = 1.56, *p* < 0.001); and family support with a 68% increase (OR = 1.68, *p* < 0.001). Conversely, each one-unit increase in social support was associated with a 37% decrease in odds (OR = 0.63, *p* < 0.001), and greater structure with a 13% reduction in odds (OR = 0.87, *p* < 0.001).

#### Relationship between resilience and psychopathology

**Figure 1:**
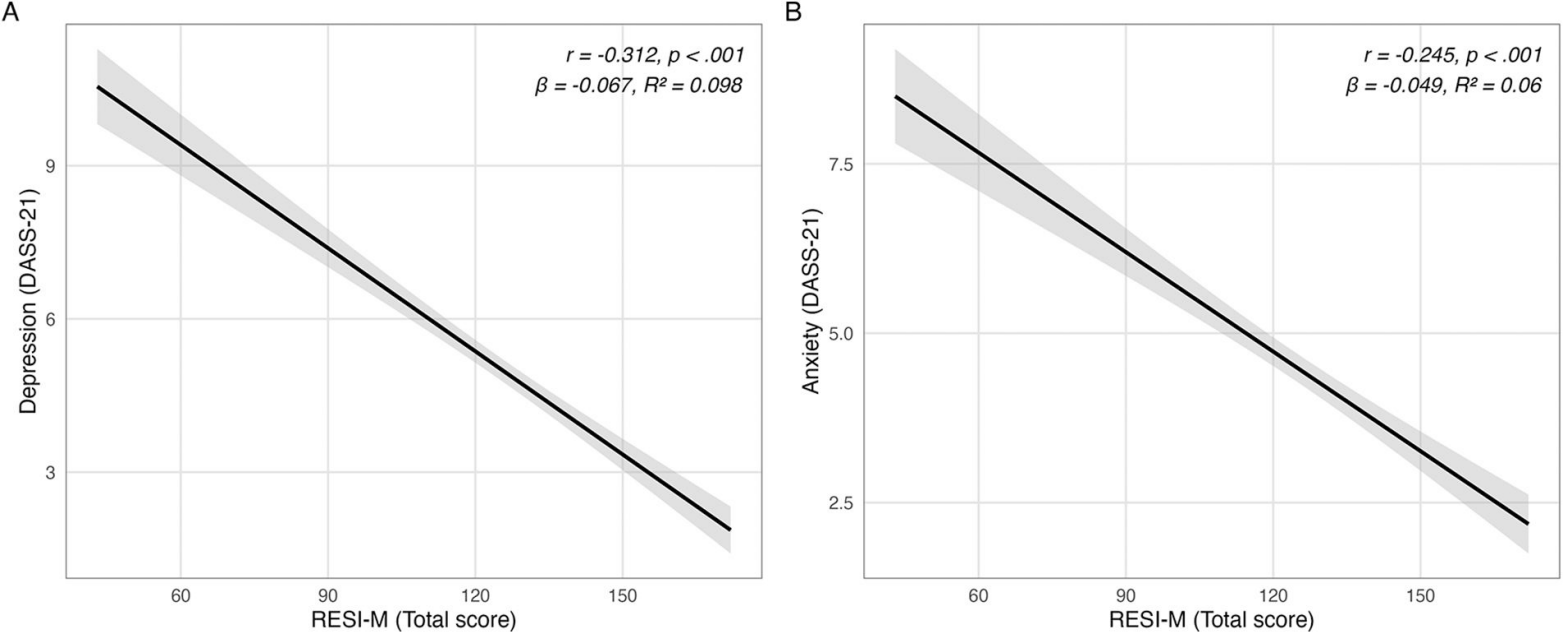
Linear regression of resilience predicting depression **(A)** and anxiety **(B)**. Shaded areas represent 95% confidence bands. Resilience significantly predicted depression (*β* = −0.067, SE = 0.004, *p* < 0.001, R² = 0.098, r = −0.312) and anxiety (*β* = −0.049, SE = 0.004, *p* < 0.001, R² = 0.060, r = −0.245). N = 2,193. ***p < 0.001..

Resilience was a significant predictor of depression, F(1, 2,192) = 237.13, *p* < 0.001, accounting for 9.8% of the variance (R² = 0.098). The unstandardized coefficient indicated that for each one-point increase in resilience, depression decreased by 0.067 points [*β* = −0.067, SE = 0.004, 95% CI (−0.076, −0.059)] (Figure 1A). A moderate negative correlation was found between resilience and depression (r = −0.312, *p* < 0.001). Resilience also significantly predicted anxiety, F(1, 2,192) = 139.68, *p* < 0.001, explaining 6.0% of the variance (R² = 0.060), with a 0.049-point decrease in anxiety per one-point increase in resilience [*β* = −0.049, SE = 0.004, 95% CI (−0.057, −0.041)]. A small negative correlation was observed for anxiety (r = −0.245, *p* < 0.001) (Figure 1B).

#### Cluster analysis and validation

##### Identification of clusters

The k-means cluster analysis revealed an optimal solution of two clusters (k = 2), as determined by the majority rule of the NbClust procedure (8 out of 30 indices proposed 2 clusters), further supported by the elbow method and average silhouette width. The characteristics of each cluster are described below:

Cluster 1 (*n* = 955, 43.5%)—Vulnerable profile. This cluster was characterized by higher scores on all psychopathology measures and lower scores on all resilience dimensions. Participants exhibited elevated depression and anxiety symptoms, corresponding to moderate-to-severe symptomatology according to DASS-21 cut-offs. Resilience scores were notably lower across all RESI-M dimensions, including social support, family support, structure, confidence, and social competence, resulting in a low total resilience score.
Cluster 2 (*n* = 1,239, 56.5%)—Resilient profile. This cluster exhibited the opposite pattern, characterized by lower psychopathology and higher resilience. Depression and anxiety scores fell within the normal range. Total resilience was substantially higher, with elevated scores across all five RESI-M dimensions. Alcohol use did not differ meaningfully between clusters.

##### Comparison between clusters

Independent-samples t-tests revealed statistically significant differences between clusters for all variables (all *p* < 0.001) (Table 4), except for alcohol use which showed a small but significant difference (*p* = 0.005). Large effect sizes were observed for most variables, with the largest differences found for total resilience [Cohen's d = −2.349, 95% CI (−2.458, −2.240)], social support [d = −2.269, 95% CI (−2.377, −2.161)], and confidence [d = −1.934, 95% CI (−2.036, −1.832)]. Depression and anxiety also showed large effects (d = 1.081 and d = 0.939, respectively). The effect size for alcohol use was very small [Cohen's d = 0.124, 95% CI (0.039, 0.208)].

**Table 4:** Comparison between vulnerable and resilient profiles.

Variable	Cluster 1 “Vulnerable profile” (*N* = 955)	Cluster 2 “Resilient profile” (*N* = 1,239)	t (df)	*p*	Cohen's d	95% CI
Depression	8 (5.8)	2.75 (3.99)	t(1,611.17) = 23.96	< 0.001	1.08	[0.99, 1.17]
Anxiety	6.94 (5.43)	2.59 (3.92)	t(1,666.58) = 20.92	< 0.001	0.93	[0.85, 1.02]
Social support	36.73 (7.18)	52.49 (6.76)	t(1,987.87) = −52.28	< 0.001	−2.26	[−2.37, −2.16]
Family support	13.82 (3.67)	19.83 (3.24)	t(1,912.79) = −39.96	< 0.001	−1.74	[−1.84, −1.65]
Structure	11.52 (2.71)	15.88 (2.63)	t(2,022) = −37.78	< 0.001	−1.63	[−1.73, −1.53]
Confidence	46.8 (10.51)	64.29 (7.73)	t(1,689.1) = −43.21	< 0.001	−1.93	[−2.03, −1.83]
Social competence	17.32 (4.34)	24.62 (4.55)	t(2,097.18) = −38.22	< 0.001	−1.63	[−1.73, −1.53]
Resilience	102.86 (18.68)	142 (14.93)	t(1,790.34) = −53.01	< 0.001	−2.34	[−2.45, −2.24]
Alcohol	2.96 (5.53)	2.31 (5.01)	t(1,943.63) = 2.84	= 0.005	0.12	[0.03, 0.20]

Values are presented as mean (standard deviation). Effect sizes (Cohen's d) interpretation: < 0.2 = very small, 0.2–0.5 = small, 0.5–0.8 = medium, > 0.8 = large.

##### Cluster distribution by geographic region

A chi-square test of independence revealed a significant association between cluster membership and geographic region, *χ*²(3) = 40.58, *p* < 0.001, with a small-to-medium effect size (Cramer's V = 0.136; Figure 1). The distribution varied across regions: Cluster 2 (resilient profile) was most prevalent in semi-rural (66.4%) and agro-commercial regions (57.9%), whereas Cluster 1 (vulnerable profile) was most prevalent in industrial urban (53.2%) and semi-urban regions (47.9%).

## Discussion

The COVID-19 pandemic and the social distancing measures implemented to mitigate its spread raised substantial concerns among parents, educators, and researchers regarding adolescents' mental health and social well-being. In this context, the present study assessed and compared the mental health status of adolescents from different regions of southern Veracruz, identifying marked regional differences in emotional symptoms, substance use, resilience and psychosocial support.

Overall, between 20% and 38% of adolescents reported alcohol consumption and exhibited symptoms of anxiety, depression, and stress, while only 21.2% demonstrated high resilience levels. These findings indicate a considerable burden of emotional distress in this population group. Importantly, both the descriptive analysis and multivariable regression models revealed significant regional disparities, underscoring depression as a consistent predictor of belonging to regions characterized by greater psychosocial vulnerability. In contrast, adolescents from Region 4 (semi-rural) showed a more favorable mental health profile, with a lower prevalence of alcohol use, anxiety, stress, and depression, as well as higher levels of resilience and its protective components.

The prevalence of alcohol and other substance use observed in this study was notably lower than that reported in previous surveys conducted in the state and surrounding regions, where lifetime alcohol use ranged from 57.4% to 71.4%, and tobacco, marijuana, inhalants, and cocaine use were substantially higher (25, 27, 33, 38). This reduction is consistent with international and national evidence documenting a decline in adolescent substance use during the pandemic, which has been associated in previous studies with social restrictions that may have limited peer interaction and access to substances which typically occurs outside the home environment (8, 9, 39). Nevertheless, these changes should be interpreted with caution, as substance use patterns may rebound following the lifting of pandemic-related restrictions, highlighting the need for continued monitoring and preventive strategies to address this issue.

Despite the reduction in substance use, emotional distress remains prevalent. Anxiety, stress, and depressive symptoms were commonly reported, aligning with previous studies indicating that social isolation, disruption of daily routines, domestic violence, excessive screen time, sedentary behavior, exposure to COVID-19–related illness, and pre-existing mental health conditions have exacerbated psychological symptoms among adolescents during the pandemic (6, 7). The prevalence of anxiety and depression observed in our sample falls within the ranges previously reported among Mexican adolescents, where anxiety rates of 48%–64% and depression rates of 59%–78% have been documented (24, 26, 40).

Given the neurobiological vulnerabilities and the chronosystemic impact of the pandemic outlined earlier, the high prevalence of substance use and emotional dysregulation observed across most regions in our sample is a significant clinical concern. Early alcohol use is known to precipitate cognitive impairments and alter neurodevelopment (41), frequently linking to the early initiation of tobacco and other drugs consumption. This cascade increases the risk of accidents, interpersonal violence, and severe psychopathology (24, 42, 43). Therefore, the comparatively low prevalence of substance use and emotional distress observed in Region 4 reflects a more favorable psychosocial profile and may be indicative of conditions associated with better mental health outcomes of adolescents in these communities.

In this context, the consistent association between depressive symptoms and regional vulnerability identified in the regression analysis is particularly concerning. Prior research has demonstrated that depression and anxiety often precede severe adverse outcomes, including suicidal behaviors. A follow-up study of individuals who died by suicide showed that approximately half exhibited anxiety symptoms shortly before death, often accompanied by depressive symptoms and substance use problems (44). These findings reinforce the importance of early identification and intervention for emotional distress during adolescence.

Our analyses consistently confirmed that resilience is a significant protective factor against adolescent psychopathology. Linear regression showed that higher resilience scores predicted lower depression and anxiety symptoms, aligning with systematic review evidence demonstrating that school-based resilience interventions effectively reduce internalizing problems and psychological distress in adolescents (45). Importantly, this inverse relationship held even during the chronic stress of the COVID-19 pandemic. The k-means cluster analysis complemented these findings by identifying two distinct psychosocial profiles: a Resilient Profile (56.5% of the sample) characterized by low symptomatology and high resilience across all dimensions, and a Vulnerable Profile (43.5%) showing the opposite pattern. The large magnitude of differences between groups, particularly in total resilience and social support, is consistent with recent studies using similar person-centered approaches. However, in contrast to studies that have identified a subgroup of depressed youth with high resilience despite high symptom burden (46), our two-cluster solution revealed a strong inverse relationship between psychopathology and resilience. Similarly, another study found differentiated family resilience patterns associated with better adaptation in adolescents with emotional disorders (47). Our study extends these inverse profiles by demonstrating that they are not exclusive to clinical populations but represent generalizable patterns of stress adaptation in community-dwelling adolescents during a collective chronic stressor like the COVID-19 pandemic. However, it is important to note that the cluster analysis reveals mutual dependency among these factors (e.g., depression, resilience, social support) within each profile, but does not establish causal direction. Therefore, our findings provide weak evidence to suggest that targeting resilience alone would necessarily alter depression or other mental health outcomes. The observed associations are consistent with the interpretation that resilience, social support, and psychopathology covary together, but causal inferences require experimental or longitudinal designs.

Notably, lifetime alcohol use did not meaningfully differ between clusters, despite literature suggesting an association between substance use and lower resilience (48). This may reflect the unique pandemic context, when social restrictions reduced substance use and emotional distress manifested predominantly through internalizing symptoms rather than externalizing behaviors. Finally, the significant association between cluster membership and geographic region underscores that psychological profiles are shaped by ecological context. The overrepresentation of the Resilient Profile in Region 4 (semi-rural, indigenous presence) and the Vulnerable Profile in Region 3 (urban-industrial) suggests that sociocultural and economic characteristics influence coping patterns. This finding supports the notion that resilience is not solely an individual attribute but emerges from interactions between the person and their environment, including family support, social cohesion, and contextual stressors (49). Exposure to childhood adversity, more prevalent in structurally disadvantaged regions, may explain why adolescents in urban-industrial areas presented a more vulnerable profile compared to those in semi-rural communities with stronger cultural roots (49).

Regarding resilience, our findings indicate that most adolescents exhibited medium-to-low levels, with only one-fifth demonstrating high resilience. Adolescents in Region 4 displayed significantly higher resilience, which may reflect more favorable caregiving environments and social conditions than in other regions. Resilience is shaped by consistent emotional support during childhood, effective emotion regulation, problem-solving skills, secure attachment, and self-efficacy, which are attributes that are strengthened through supportive family and social environments (50, 51). The higher resilience observed in Region 4 is consistent with the lower prevalence of depressive symptoms and substance use.

It is important to recognize that the association between protective factors, such as family support networks, and improved mental health outcomes is well established in the literature, not only in adolescence but throughout the lifespan (14, 50, 51). However, the distinctive contribution of this study lies not in confirming this general relationship but in elucidating how the regional sociocultural context modulates the effectiveness of these protective factors during a global health crisis. Our data reveal that while support is universally beneficial, its presence and impact vary significantly according to community structure. For example, Region 4, characterized by semi-rural environments and an Indigenous presence, exhibited significantly higher levels of family support and structure than urban industrial regions, despite having less infrastructure. These findings suggest that region-specific social and cultural characteristics may help create a more protective psychosocial environment, shifting the debate from identifying protective factors to understanding their contextual determinants.

Social and family support emerged as key protective factors in both descriptive analyses and regression models. In contrast to resilience, which may be more trait-like or slower to change, social support is a modifiable factor that can be directly targeted through interventions. The cluster analysis supports this interpretation, as social support showed the second largest effect size between profiles (Cohen's d = −2.27), just after total resilience. Adolescents facing socially vulnerable conditions benefit substantially from support provided by family members, peers, teachers, and community institutions, which can buffer the effects of adversity, such as violence, poverty, and limited institutional resources (50, 51). Nevertheless, the directionality of these relationships remains to be tested in future intervention studies. The protective role of social support is particularly relevant given the well-documented association between low resilience and higher levels of depression, anxiety, and alcohol consumption (14, 17). Strengthening resilience has been shown to reduce the negative consequences associated with socially motivated alcohol use, suggesting that resilience-building interventions may serve as effective preventive strategies (16, 17).

The comparatively favorable profile observed in Region 4 (characterized by higher resilience, lower depressive symptoms, and greater family and social support), supports ecological models of adolescent mental health, which emphasize the interaction between individual characteristics and broader social systems, including family, culture, and socioeconomic context. These findings align with those of previous studies showing that resilience and family support mitigate the impact of academic stress and psychological distress among adolescents and young adults (13–17, 50, 51).

While demographic data indicate that this semi-rural region has less formal school infrastructure than industrialized urban centers, our regression models identified “Family Support” and “Structure” as significantly associated predictors for this region. This suggests that in contexts of less industrial integration and a greater indigenous presence, culturally rooted social structures may act as protective factors for public health, compensating for the scarcity of institutional resources.

This finding aligns with previous evidence indicating that adolescents in socially vulnerable situations benefit substantially from the support provided by family members and community institutions, which mitigates the effects of adverse experiences (50, 51). Furthermore, the resilience observed in this marginalized population reflects their capacity to adapt to adversity, where community cohesion and local cultural practices (such as the use of indigenous languages and subsistence farming) are associated with a stronger sense of belonging and social structure. Therefore, the implications for other regions do not necessarily lie in replicating material resources but rather in strengthening the social and family support networks that proved to be crucial in this context.

This study has several limitations. The present study focused exclusively on public high school students, which limits the generalizability of the findings to adolescents attending private institutions or those not enrolled in formal education. The data were collected in 2021, and the cross-sectional design of the study precludes drawing causal inferences and may not fully reflect post-pandemic changes. Therefore, the observed associations should be interpreted as contextual correlations rather than direct protective mechanisms. The reliance on self-reported questionnaires introduces the potential for recall or social desirability bias, and non-probabilistic sampling further restricts the extent to which results can be generalized beyond the study regions. Future research should incorporate longitudinal designs, mixed-methods approaches, and objective measures to effectively capture the dynamics of mental health and resilience. Furthermore, examining family dynamics, socioeconomic conditions, school environments, and community resources would facilitate a more profound comprehension of regional disparities. Future research should examine whether the relationship between resilience and mental health outcomes is moderated by regional context.

Despite these limitations, our findings provide valuable evidence for informing public health policies and intervention strategies. Programs aimed at strengthening family and social support networks, enhancing resilience, and addressing depressive symptoms may be particularly effective if they are tailored to regional contexts. Existing evidence-based interventions that target social support in adolescents include school-based mentoring programs (e.g., Big Brothers Big Sisters), family strengthening interventions (e.g., the Strengthening Families Program), and group-based social skills training. These interventions have demonstrated effectiveness in reducing depressive symptoms and improving resilience (52–55). Future research should examine whether regionally tailored versions of these interventions could reduce the disparities observed in the present study. Health professionals, educators, and policymakers should prioritize culturally sensitive community-based interventions to promote adolescent mental health and reduce psychosocial vulnerability in southern Veracruz.

## Conclusion

The present study documents a high prevalence of emotional distress and psychoactive substance use among adolescents in southeastern Veracruz, accompanied by generally low levels of resilience. Substantial regional disparities were identified, with Region 4 (semi-rural) showing a more favorable profile, including lower levels of depressive symptoms and higher levels of family and social support. Resilience consistently protected adolescents against depression and anxiety. Importantly, cluster analysis showed that resilient profiles were more common in semi-rural Region 4, whereas vulnerable profiles predominated in industrial urban Region 3 (industrial urban), underscoring how regional context shapes mental health outcomes.

These findings highlight the importance of considering regional context in assessing adolescent mental health and provide an empirical basis for developing targeted prevention and intervention strategies. Family and social support emerged as key modifiable protective factors for resilience-building interventions. While the study was conducted during the COVID-19 pandemic, the cross-sectional design does not allow causal inferences regarding the origin of the observed patterns. Nevertheless, the results suggest that sociocultural and contextual factors may be associated with differences in mental health outcomes, underscoring the importance of incorporating local social dynamics into public health approaches to improve adolescent well-being.

## Data Availability

The raw data supporting the conclusions of this article will be made available by the authors, without undue reservation.
