# Supplementary material for: Socio-cultural context as a protective factor: regional disparities in adolescent mental health in Mexico during the COVID-19 pandemic
Source: Front Child Adolesc Psychiatry. 2026 Jun 22;5:1783057. doi: 10.3389/frcha.2026.1783057 (PMC13333678; doi:10.3389/frcha.2026.1783057)
Supplement: Supplementary file 1 [file Table1.docx]

*Attached tables*

*Table 1a:* Severity levels of stress, anxiety, depression, and resilience by region

|  |  | **Regions** | | | | | |
| --- | --- | --- | --- | --- | --- | --- | --- |
|  |  | **I** | **II** | **III** | **IV** | **Total** | **Cramer´s V** |
| **Stress** | *Mild* | 642 (72.7) | 369 (78.0) | 179 (68.1) | 479 (83.3) | 1669 (76.1) | 0.08* |
|  | *Moderate* | 105 (11.9) | 43 (9.1) | 28 (10.6) | 41 (7.1) | 217 (9.9) |  |
|  | *Severe* | 110 (12.5) | 51 (10.8) | 50 (19.0) | 39 (6.8) | 250 (11.4) |  |
|  | *Extremely severe* | 26 (2.9) | 10 (2.1) | 6 (2.3) | 16 (2.8) | 58 (2.6) |  |
| **Anxiety** | *Mild* | 507 (57.4) | 306 (64.7) | 134 (51.0) | 403 (70.1) | 1350 (61.5) | 0.08* |
|  | *Moderate* | 112 (12.7) | 55 (11.6) | 48 (18.3) | 62 (10.8) | 277 (12.6) |  |
|  | *Severe* | 69 (7.8) | 34 (7.2) | 20 (7.6) | 26 (4.5) | 149 (6.8) |  |
|  | *Extremely severe* | 195 (22.1) | 78 (16.5) | 61 (23.2) | 84 (14.6) | 418 (19.1) |  |
| **Depression** | *Mild* | 544 (61.6) | 326 (68.9) | 160 (60.8) | 424 (73.7) | 1454 (66.3) | 0.08* |
|  | *Moderate* | 128 (14.5) | 60 (12.7) | 28 (10.6) | 73 (12.7) | 289 (13.2) |  |
|  | *Severe* | 97 (11.0) | 33 (7.0) | 29 (11.0) | 24 (4.2) | 183 (8.3) |  |
|  | *Extremely severe* | 114 (12.9) | 54 (11.4) | 46 (17.5) | 54 (9.4) | 268 (12.2) |  |
| **Resilience** | *Low* | 297 (33.6) | 155 (32.8) | 88 (33.5) | 118 (20.5) | 658 (30.0) | 0.10* |
|  | *Medium* | 424 (48.0) | 217 (45.9) | 132 (50.2) | 297 (51.7) | 1070 (48.8) |  |
|  | *High* | 162 (18.3) | 101 (21.4) | 43 (16.3) | 160 (27.8) | 466 (21.2) |  |

Data are presented as n (%). Differences across regions were assessed using the chi-square test. Effect size is reported as Cramer’s V. p < 0.05.
